# Controversies and advances in the management of congenital ptosis

**DOI:** 10.1586/17469899.2015.991389

**Published:** 2014-12-12

**Authors:** Ali Mokhtarzadeh, Andrew R Harrison

**Affiliations:** ^a^ Department of Ophthalmology and Visual Neurosciences, University of Minnesota, MMC 493, 420 Delaware St, SE, Minneapolis, MN, USA; ^b^ Department of Otolaryngology and Head and Neck Surgery, University of Minnesota, Minneapolis, MN, USA

**Keywords:** blepharoptosis, childhood ptosis, congenital ptosis, frontalis sling, pediatric ptosis

## Abstract

The management issues associated with pediatric ptosis begin with determining the etiology of the ptosis, and considering how the eyelid position affects the child’s visual and psychosocial development. These ultimately determine if and when surgical management should be undertaken. Surgical challenges include the lack of intraoperative feedback regarding the dynamic eyelid height and contour under general anesthesia. When the eyelid elevators do not function or if there is little drive to lift the involved eyelid, obtaining good surgical outcomes can be extremely challenging. A plethora of surgical techniques and materials have been developed, each with their own benefits and drawbacks. Careful preoperative evaluation, planning and counseling can usually result in satisfactory surgical results with happy parents and patients. Families should always be aware that the child will need to be followed long term for visual development, ocular health, and they need to be counseled regarding the possibility of revision surgery.

Pediatric congenital ptosis, while commonly encountered by the pediatric ophthalmologist and oculofacial plastic surgeon, continues to be a challenging management issue. The challenges begin with the evaluation of a preverbal child in the presence of anxious parents. Any red flags need to be teased out, and a decision regarding ptosis repair is made. Timing of ptosis repair or intervals for follow-up become another issue. Finally, if surgical repair is decided upon, there are numerous surgical techniques available, each with unique advantages and drawbacks. Special consideration needs to be given to the risk of amblyopia as well as psychosocial development of the child. In recent decades, numerous modifications have been described to the classic procedures in an effort to improve surgical outcomes.

## Background

Data about the incidence of childhood ptosis are limited, with a population-based study out of Olmsted County, Minnesota, finding an incidence of 7.9 per 100,000 children under the age of 19. Of these, 89.7% were congenital, of which 84.3% were diagnosed as simple congenital ptosis [1].

### Categorization of childhood ptosis

Childhood ptosis can be classified as aponeurotic, myogenic, neurogenic, mechanical and pseudoptosis. A multifactorial ptosis can also be encountered.

*Aponeurotic ptosis*, most commonly associated with adult ptosis, suggests a normal levator palpebrae superioris muscle, with normal function. A diaphanous tendon is unable to translate the contraction of the muscle to complete elevation of the eyelid. In children, this form of ptosis can be seen with birth or other trauma, or with chronic contact lens wear.

*Myogenic ptosis*, which is the most common etiology of congenital ptosis, indicates a congenital abnormality of the levator muscle itself. Injury to the eyelid *in* *utero* can cause a secondary myogenic ptosis, such as can be seen with amniocentesis. Introperatively, these muscles tend to look fibro-fatty infiltrated and are quite inelastic [2]. This results in both an inability to lift the eyelid and retraction of the eyelid in downgaze and lagophthalmos. Non-congenital forms of myogenic ptosis include muscular dystrophy and chronic progressive external ophthalmoplegia, and while they are classically encountered in adulthood, they may occasionally manifest during childhood.

*Neurogenic ptosis* most commonly results from an abnormality of the oculomotor nerve as it supplies the levator palpebrae muscle or a problem with sympathetic innervation of Muller’s muscle. This can be congenital or acquired. Synkinetic syndromes can also be classified as neurogenic. Every examination should include thorough evaluation of pupils and ocular motility. Aberrant regeneration, such as elevation of the ptotic lid with depression, abduction or adduction, is highly suggestive of acquired oculomotor paralysis. Pupil exam should not only consist of size and reaction, but iris heterochromia should also be noted, which would suggest a congenital Horner syndrome. Myasthenia gravis also rarely presents in childhood as acquired ptosis.

**Figure 1.**
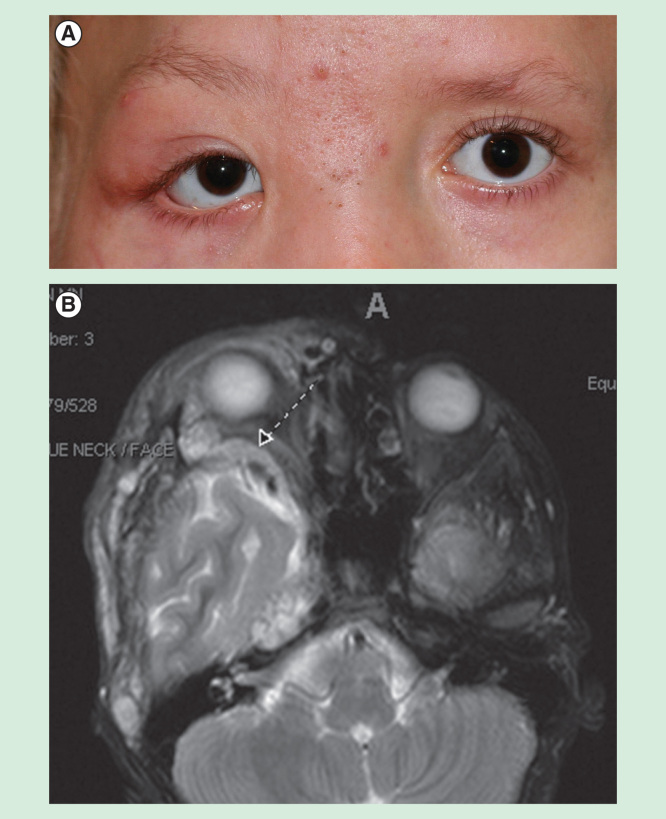
**A 7-year-old child with neurofibromatosis.** She has a dysplastic greater wing of the sphenoid on the right, and a neurofibroma involving the right superior orbit and upper eyelid as seen on imaging.

*Mechanical ptosis* suggests the eyelid is unable to be opened secondary to a mass or being tethered downward. In children, this can be due to brow ptosis, a mass, infiltration, inflammation, foreign body, adhesions and cicatrix. Classic masses causing mechanical ptosis include neurofibroma (Figure 1) and capillary hemangioma.

*Pseudoptosis* can be a term used for ‘other’ etiologies of apparent ptosis. This can be secondary to enophthalmos, microphthalmos, anophthalmos, phthisis or contralateral eyelid retraction.

Numerous craniofacial syndromes and cranial dysinnervation disorders can be associated with childhood ptosis. Classic syndromes associated with congenital or childhood ptosis, often requiring greater thought in management and surgical planning, include Duane retraction syndrome, blepharophimosis, congenital fibrosis of the extraocular muscles and Marcus Gunn jaw-winking syndrome. While discussion of each of these individual syndromes is beyond the scope of this manuscript, every surgeon managing childhood ptosis should be familiar with them and the challenges they each present in management.

### Preoperative considerations

The inability of the very young to cooperate as well as anxious parents can complicate the evaluation of the ptotic child. Decision to operate needs to take into consideration whether the eyelid position is amblyogenic, responsible for a chin-up position and how the eyelid position may affect the child’s social interactions. In the setting of amblyogenic ptosis, there is little question as to the appropriate timing of ptosis surgery. Controversy exists for timing of non-amblyogenic ptosis. Many surgeons decide to proceed with surgical correction prior to the child beginning kindergarten in an effort to prevent comments from peers that may make the child more self-conscious. Waiting until age 4 or 5 allows for a more cooperative examination. Others advocate a more aggressive approach with early surgery typically around the age of 1, arguing that enough clinical information can be ascertained at that age, that there is more emotional trauma associated with surgery later in childhood and that children may encounter hurtful comments while playing with other children prior to kindergarten [3].

Other salient preoperative considerations include whether the child has the drive to recruit frontalis to compensate for the ptotic eyelid or if they are completely ignoring it, variability of eyelid position, presence of strabismus and synkinetic movements. If there is no frontalis recruitment on the ptotic side in a child with poor function ptosis, frontalis sling procedures become less predictable, and one may consider supramaximal levator resection in their place. The author’s preference, in this setting, is resection of 2–3 mm of tarsus and 4–6 mm of conjunctiva and Muller’s muscle, in addition to levator advancement. If there is apparent unilateral ptosis, consideration should be given to the presence of asymmetric ptosis, which frequently occurs but is masked by a strong central drive to open the ptotic eyelid, and aggressive frontalis recruitment; such masked cases will become evident in the postoperative period when the strong drive to raise the more ptotic eyelid is lost.

As with adults, making note of the marginal reflex distance (MRD1) is important, but of paramount importance in surgical planning is the degree of upper eyelid excursion, often referred to as levator function. This is the difference between the position of the upper eyelid in downgaze and upgaze, with the brow immobilized. Beard categorized levator function as good, fair or poor (Table 1) [4]. We consider >14 mm of function as normal. Determining the response of the eyelid position to instillation of phenylephrine drops (10 or 2.5%) [5] is helpful in planning an appropriate procedure [6,7]. Other subtleties should be evaluated, including how much effort the child exudes to utilize the involved eye, presence or absence of the Bell’s phenomenon (elevation and slight abduction of the eye on attempted lid closure) and the strength of the eyelid crease. While children generally tolerate corneal exposure better than adults, in the absence of a good Bell’s phenomenon, the authors prefer to be less aggressive surgically to minimize the risk of postoperative corneal complications.

**Table 1.** **Beard's classification of levator function.**

**Levator function**	**Eyelid excursion**
Good	8–16 mm
Fair	5–7 mm
Poor	4 mm or less

Finally, preoperative counseling and setting expectations is crucial. Parents should understand that the eyelids may never be exactly symmetric, that there may be complications associated with the surgery and that there is a relatively high rate of revisions. In one large series of 186 eyes of 155 children, there was a 19.89% revision rate, with a further 9.14% who declined further revision [8]. Additionally, the parents should be aware that as the child grows, despite their eyelid surgery, they might need to be followed for refractive error and amblyopia.

### Surgical procedures

Determining an appropriate procedure should be individualized, and obtaining ideal surgical outcomes can be both challenging and controversial. One can classify surgical procedures anatomically or based on what degree of ptosis they can be utilized for. We will base our discussion on levator function. We consider good levator function 10 mm or greater excursion, moderate 7–10 mm, fair 5–7 mm and poor less than 5 mm of excursion. It must be noted that numerous procedures and modifications have been described and continue to be utilized; the below only addresses some of the most commonly used.

#### Good levator function

Over the past decade, the use of 2.5% phenylephrine ophthalmic drops in preoperative evaluation of children with relatively good levator function has become routine in our practice. The addition of Muller’s muscle conjunctival resection, with or without superior tarsectomy, to the armamentarium of procedures to address pediatric ptosis has allowed for quite predictable and pleasing results in children whose eyelid elevates in response to the drop. There are numerous nomograms, and the amount of resection should be tailored and modified based on the surgeon’s personal results. Our current practice is to resect 9 mm of conjunctiva and Muller’s muscle if the elevation is perfect. The amount of resection can be anywhere between 6.5 and 9.5 mm based on the degree of ptosis and response to phenylephrine. Superior tarsectomy can be added to the procedure, to provide an additional 1–2 mm of lift. Our current practice is to resect 1 mm of additional tarsus for each 1 mm of desired additional lift. Muller’s muscle conjunctival resection is not considered a repeatable procedure. Critics of the procedure argue resecting normal conjunctiva will worsen dry eye by removing goblet cells, and that it does little to address the anatomical problem, which is with the levator palpebrae muscle [9]. In our hands, we have not had trouble with dry eyes in the pediatric population, and the predictable outcome without intraoperative adjustment and pleasing eyelid contour has made this our procedure of choice in the setting of mild congenital ptosis, with good response to phenylephrine drops.

Utilization of the Fasanella–Servat procedure [10] also has a role in management of up to 2.5 mm of congenital or acquired ptosis. This has become a favored procedure for children with mild degrees of ptosis who have minimal response to phenylephrine or who have mild residual ptosis following prior Muller’s muscle conjunctival resection. The ratio of tarsectomy to eyelid elevation is 2 mm of tarsectomy to 1 mm of desired elevation [11]. Given most children require general anesthetic, Muller’s muscle conjunctival resection and Fasanella–Servat procedure allow graded surgery based on preoperative measurements rather than intraoperative observations, which may be skewed by local anesthetic and the depth of anesthesia. Children with congenital ptosis frequently have a poor eyelid crease, and this may limit the aesthetic result even if the eyelid height and contour is perfectly symmetrical. The Fasanella–Servat and Muller’s muscle conjunctival resection procedures do little to create an eyelid crease.

#### Fair to good levator function

While Muller’s muscle surgery has become our preferred workhorse for mild degrees of congenital ptosis, levator surgery is necessary for those with fair levator function, good levator function and inadequate response to phenylephrine, or those who have previously undergone resection of Muller’s muscle and/or tarsus and require further revision.

With the added benefit of allowing creation or accentuation of a lid crease, a significant drawback of all procedures other than Muller’s muscle surgery is the question of where to position the eyelid under general anesthesia. While those with good levator function typically require advancement of the levator aponeurosis, in cases of moderate or fair levator function, levator resection also becomes necessary. Beard described a method in which the amount of levator resection is based on the degree of ptosis and eyelid excursion [12]. This can be difficult to incorporate into any given surgeon’s practice as the amount resected is influenced by surgical technique and tension on the levator muscle. Berke provided guidelines on where to set the upper eyelid under general anesthesia based on upper eyelid excursion [13], and there have been minor modifications since (Table 2) [14].

**Table 2.** **Intraoperative eyelid height under general anesthesia based on upper eyelid excursion for levator surgery.**

**Levator function**	**Lid position on cornea**
>10 mm	3–4 mm below the limbus
8–9 mm	3 mm below the limbus
6–7 mm of function	2 mm below the limbus
5–6 mm of function	1 mm below the limbus
0–4 mm of function	Recommend frontalis suspension

Adapted from [14].

#### Poor levator function

The most source of controversy surrounding congenital ptosis is in the management of children with poor levator function. While some advocate supramaximal levator resection in the setting of poor levator function, we have found utilization of a frontalis sling to yield the most satisfactory results. The material used and technique for placement of a frontalis sling continues to evolve.

**Figure 2.**
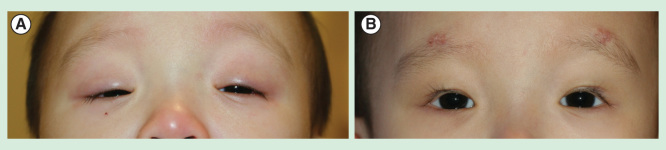
**Poor function ptosis. (A)** An 8-month-old child with severe bilateral ptosis, which had resulted in a significant chin-up position. **(B)** One week after bilateral upper eyelid ptosis repair with silicone rod frontalis sling.

Autogenous tensor tendon fascia lata, while considered the gold standard, carries the disadvantage of a second surgical site. Furthermore, the tendon is incompletely developed in young children and should be avoided in children younger than 4 years. While banked fascia is available, we have transitioned almost exclusively to utilization of synthetic materials and silicone in particular. Available synthetic suspension materials include nylon, polyester, polytetrafluoroethylene, polypropylene and silicone. Ben Simon *et al*. reviewed their experience with a variety of autologous, banked and synthetic materials and found their recurrence rate of ptosis to be 26% at a mean of 12 months, with no difference between suture materials or loop shape [15]. This supports the widely held belief that ultimately it is scar tissue that surrounds the suspension material that is responsible for transferring lift from the frontalis to the eyelid. Currently, we have been most satisfied with the characteristics of silicone as the suspension material (Figure 2). Advantages include its elasticity, theoretically allowing for more dynamic movement of the lid compared to stiff materials; it is adjustable, often through opening a single brow incision, and can be easily removed should problems arise.

Complications associated with frontalis sling surgery include exposure keratopathy, recurrence of ptosis and irregular contour of the lid. Often, patients will be unhappy with the surgical outcome despite excellent lift if an eyelid crease is not created or is asymmetric. Additionally depending on the vector of forces, brow recruitment may lead to undesirable peaking of the eyelid or elevation of the lid off of the globe. Use of non-autogenous materials can increase the risk of pyogenic granuloma formation, inflammatory response to implanted materials as well as infection. Exposure of sutures or sling material through the conjunctiva can result in a chronic conjunctivitis, which may initially be mistaken for exposure keratopathy.

Even when placement of a frontalis sling results in excellent symmetry in forward gaze, the lid tends to hang up in downgaze. Additionally when a child is amblyopic on the ptotic side, there may be little drive to recruit frontalis to engage the suspended eyelid. To overcome this, Beard suggested disinsertion of levator on the uninvolved side and placement of bilateral slings [16]. Others have advocated bilateral sling procedures without extirpation of levator [17], often referred to as the ‘Chicken Beard’ procedure. While this overcomes some of the issues associated with asymmetry, it is difficult for parents to agree to operate on a normal eyelid. Furthermore, operating on the normal side does have inherent risk, particularly if the side with ptosis is densely amblyopic, severe exposure keratopathy or infection on the normal side could be devastating. As mentioned earlier, in the setting of no frontalis recruitment on the side of the ptotic eyelid, if there is some levator function, the author’s preference is to combine levator advancement with resection of 2–3 mm of tarsus and 4–6 mm of conjunctiva and Muller’s muscle. Ultimately, many surgeons have found the cosmesis in primary gaze with unilateral frontalis suspension for unilateral ptosis to be the most acceptable approach to both the surgeon and patient [18,19].

## Expert commentary & five-year view

While surgical technique and materials have continued to evolve over the decades, a better understanding of the embryology and molecular genetics of how a child is born with ptosis may lead to the most radical changes in how congenital ptosis, as well as other craniofacial anomalies are approached in the future. Vestal *et al*. reviewed the literature for cases of congenital ptosis in monozygotic twins and found a heritability index of 0.75 (0–1), indicating a strong genetic contribution to the phenotype of congenital ptosis [20]. Since thesn, an autosomal dominant gene for isolated congenital ptosis was mapped to a 3-cM region in 1p32-p4.1, utilizing a large pedigree, and additional loci have been identified on chromosome 8, 14 as well as the X chromosome [21–23]. It remains to be seen how these and other candidate genes, in combination with environmental factors, result in congenital ptosis, and perhaps this understanding will allow more tailored approaches to the management of children with ptosis.

## Notes

Key issues

- Congenital ptosis refers to a lower-than-expected upper eyelid height noted shortly after birth.
- Congenital ptosis can be unilateral or bilateral.
- There is a high heritability index among monozygotic twins, indicating a strong genetic contribution.
- Congenital ptosis can be isolated or associated with other ocular, craniofacial or systemic syndromes.
- Whether or not to repair the ptosis, and its timing, is dependent on the degree of ptosis, visual and psychosocial impact.
- The appropriate surgical procedure is dependent on the degree of ptosis, levator function, presence of synkinesis, surgeon comfort and outcomes with a variety of techniques and materials.
